# Vagal Nerve Stimulation for Depression: A Review of Side Effects in Unipolar and Bipolar Disorders

**DOI:** 10.1192/j.eurpsy.2025.1351

**Published:** 2025-08-26

**Authors:** K. Noor, M. Baig

**Affiliations:** 1 Psychiatry, PsychCare Consultants Research, Ballwin, United States

## Abstract

**Introduction:**

Vagal Nerve Stimulation (VNS) therapy has emerged as a promising treatment for individuals suffering from treatment-resistant unipolar and bipolar depression. Initially approved for epilepsy, VNS therapy’s potential to improve mood symptoms in patients unresponsive to conventional treatments like antidepressants, psychotherapy, ECT, and TMS has garnered growing interest in psychiatry. This systematic review provides a comprehensive overview of the side effects associated with VNS therapy. It aims to offer a balanced perspective on the therapy’s safety profile, aiding informed decision-making for depression.

**Objectives:**

1. To identify the most common side effects of VNS therapy.

**Methods:**

A literature search was conducted within 2 databases (PubMed and Google Scholar). The PubMed search employed MeSH terms as follows: ((vagus nerve stimulation) OR (vagal nerve stimulation)) AND ((major depressive disorder) OR (MDD) OR (depression) OR (unipolar depression) OR (bipolar depression)) AND (side effects). Google Scholar search utilized Boolean operators as follows: “vagus nerve stimulation for depression” “adverse effects” “side effects.” Tools that were native to the browsers of these respective databases were used to apply the following filters: publication date (2019-2014), free full text, English, no abstracts, no miscellaneous documents and the remaining results were reviewed independently by 2 investigators to screen for the remaining inclusion and exclusion criteria. Specifically, studies were only included if they reported on the side effects of VNS therapy for depression. Studies were excluded if they did mention the side effects, or if they were not accessible to the investigators due to pay-walls, for example.

**Results:**

A total of 380 articles were identified from searching in both databases, and screening with application of our inclusion and exclusion criteria left 14 articles to be considered. The side effects of VNS therapy can range from mild and transient to more significant, and most frequently mentioned side-effects included: headache, local skin irritation, intolerable pain, and voice alteration (Jung 2023). Headache and dizziness was common for all forms of VNS, while local skin irritation was far more common in non-invasive methods of VNS involving electrodes, and serious complications from damage to the vagus nerve resulting in arrhythmias and aspiration (Bruer 2022) potentially impacting the overall quality of life and adherence to treatment.

**Conclusions:**

Vagus Nerve Stimulation (VNS) offers a promising treatment option for patients with treatment-resistant depression, providing symptom relief when other therapies fail. However, side effects, particularly laryngeal, and variability in patient response highlight the need for personalized approaches. Future research on transcutaneous VNS and optimized protocols is essential to enhance outcomes.

**Disclosure of Interest:**

None Declared

